# Tac2-N acts as a novel oncogene and promotes tumor metastasis via activation of NF-κB signaling in lung cancer

**DOI:** 10.1186/s13046-019-1316-7

**Published:** 2019-08-30

**Authors:** Xianglin Hao, Li-yun Gao, Ning Zhang, Hongqiang Chen, Xiao Jiang, Wenbin Liu, Lin Ao, Jia Cao, Fei Han, Jinyi Liu

**Affiliations:** 1 Institute of Toxicology, College of Preventive Medicine, Third Military Medical University, 30 Gaotanyan Street, Shapingba District, Chongqing, 400038 People’s Republic of China; 2 School of Public Health, Xinxiang Medical University, Xinxiang, People’s Republic of China; 3 Cooperative innovation center of molecular diagnosis and medical inspection technology, Beijing, People’s Republic of China

**Keywords:** Tac2-N, Metastasis, NF-κB signaling pathway, Lung cancer

## Abstract

**Background:**

High rates of recurrence and metastasis are the major cause of the poor outcomes for patients with lung cancer. In previous research, we have demonstrated that Tac2-N promotes tumor growth by suppressing p53 signaling in lung cancer. Beyond that, other biological functions and clinical significance of Tac2-N in lung cancer progression are still unknown.

**Methods:**

Tissue microarrays of 272 lung cancer patients were constructed to assess the association of Tac2-N expression and prognosis of lung cancer patients with different clinical stages. The protein expression of Tac2-N in metastatic and non-metastatic specimens were detected by IHC. In vitro migration and invasion and in vivo nude mice metastasis model were used to evaluate the effect of Tac2-N ectopic expression on metastasis capability of lung cancer cells. The downstream signaling pathway of Tac2-N was explored using luciferase reporter assays and WB.

**Results:**

The expression of Tac2-N was associated with advanced stages, but not with early stages (*P* = 0.513). Tac2-N expression is sharply overexpressed in metastatic tumors compared with non-metastatic tumors. In vitro and in vivo assays suggested that Tac2-N facilitated migration and invasion of lung cancer cells in vitro and promoted tumor metastasis in vivo. Mechanistically, Tac2-N increased the degradation of IκB by promoting its phosphorylation, and subsequently activated NF-κB activity by facilitating the nuclear translocation of NF-κB and stimulating the transcription of targets, MMP7 and MMP9. Notably, the C2B domain of Tac2-N was crucial for Tac2-N to activate NF-κB signal. Blockage of NF-κB by shRNA or inhibitor attenuates the function of Tac2-N in the promotion of metastasis.

**Conclusions:**

Our study provided proof of principle to show that Tac2-N serves as a novel oncogene gene and plays an important role in the progression and metastasis of lung cancer.

**Electronic supplementary material:**

The online version of this article (10.1186/s13046-019-1316-7) contains supplementary material, which is available to authorized users.

## Background

Lung cancer is one of the global public health problems with high morbidity and mortality, and tumor metastasis persists as a main barrier to successful cancer therapy and as a significant contributor to death in patients with lung cancer [1, 2]. Metastasis requires invasive tumor cells to move from the primary neoplasm to a distant tissue, comprising a multistep process [3]. The NF-κB family of transcription factors is of particular interest in cancer metastasis, because it can regulate transcription of target genes that promote invasion and metastasis [4–6]. However, the regulatory mechanism of NF-κB signaling pathway is remained to be explored in detail. Thus, identification of new upstream regulators of NF-κB signal is of great importance for prognosis prediction and therapy.

The C2 domain is independently folded modules, of about 130 residues, found in a large and diverse set of eukaryotic proteins [7]. This domain is originally identified as cellular Ca^2+^ effectors and is found in various signaling molecules and proteins involved in vesicular tracking [7–9]. The following study manifests that the C2 domains of a variety of proteins also play a pivotal role in cellular signal transduction, protein-protein interactions and tumorigenesis [10]. For example, the C2 domain of Smurf1 directly interacts with the kinase domain of PIPKIγ and regulates cell growth and migration of lung cancer [11]. Myoferlin is involved in regulation of cellular lipid metabolism and promotion of metastases in triple-negative breast cancer [12]. NEDD4L is underexpressed in colorectal cancer and suppresses Wnt signaling pathway [13].

Tandem C2 domains nuclear protein (Tac2-N), located on human chromosome 14q32.12, encodes a putative C2 domain-containing protein that belongs to the carboxyl-terminal type (C-type) tandem C2 protein family [14]. It was originally cloned from mouse and identified contains two C-terminal C2 domains, C2A domain and C2B domain. According to previous results, Tac2-N can regulate cell proliferation in vitro and tumor growth in vivo [15]. However, the mechanisms roles and molecular mechanisms of Tac2-N in progression and metastasis of lung cancer are still unclear.

In this study, we identified Tac2-N as a novel tumor metastasis-related gene in human lung cancer through tissue microarray analysis. We evaluated the prognostic significance of Tac2-N in patients with different clinical stages, and found that Tac2-N expression was an independent poor prognostic factor for advanced stage patients, but not for early stage patients. Further, we demonstrated that the expression of Tac2-N is correlated to metastasis status of patients. Subsequently, through in vitro and in vivo metastasis assays, Tac2-N have been considered as a metastatic promoter in lung cancer. The further mechanism research showed that Tac2-N exerted its pro-tumor function through activating NF-κB signaling in lung cancer.

## Methods

### Cell lines

The lung cancer cell lines (H1975 and H1299) were purchased from the Cell Bank of the Chinese Academy of Science (Shanghai, China) and maintained at 37 °C with 5% CO2. All lung cancer cell lines were cultured in RPMI 1640 mediums (Gibco, CA). A total of 10% fetal bovine serum (FBS) was supplemented in the culture medium.

### Tissue microarray (TMA) analysis

Tissue microarrays contained 272 lung tumor samples and 265 adjacent non-tumorous lung tissues were obtained from the collaboration (Shanghai Biochip Co Ltd., Shanghai, People’s Republic of China) with the agreement of the patients. IHC staining was performed using a rabbit polyclonal antibody against Tac2-N (1:500; Abcam). The quantitative methods for evaluating protein expression of Tac2-N are described in a previous study [15]. All subjects signed an informed consent form. This study was approved by the ethics committee of Third Military Medical University. Moreover, written consent was received from patients.

### Plasmid construction and cell transfection

For overexpression, the full-length open reading frame of human Tac2-N or truncation were generated by synthesis and subsequent molecular cloning into pIRES2-EGFP. For knockdown, two DNA fragments encoding the hairpin precursors for Tac2-N were synthesized, and then inserted into shRNA expression vector GV248. Cells were transfected using Lipofectamine2000 Reagent (Invitrogen Preservation, Carlsbad, CA, USA) according to the manufacturer’s instructions. The stably transfected cells were screened under G418 (Calbiochem, La Jolla, CA, USA) or Puromycin (Sigma). Cell clones were obtained by the limited diluted method. NF-κB pathway reporter plasmid pNF-κB-TA-luc was purchased from Beyotime Biotechnology (Jiangsu, China).

### RNA isolation and qRT–PCR analysis

Total RNA was extracted using Trizol reagent (Invitrogen, Life Technologies). The cDNA was synthesized from 3 μg of total RNA using the Reverse Transcription System (Promega, Madison, WI, USA) according to the manufacturer’s instructions. All qRT-PCR reactions were performed using the C1000 Real-Time Cycler (Bio-Rad Laboratories, Hercules, CA, USA) and qRT-PCR Master mixes (Promega, Madison, WI, USA). Primers for amplification of the MMP7, MMP9, p65, p50 and ACTIN genes are listed in Additional file 1: Table S1. Expression of target genes was determined according to the 2^-ΔΔt^ method using ACTIN as a reference gene. All experiments were carried out in triplicate.

### Luciferase reporter assay

The cells (*n* = 5 × 10^4^) were grown in 24-well plates in triplicate for each condition and transfected with NF-κB pathway reporter together with indicated plasmids. At 24 h after transfection, the cells were harvested and lysed. Luciferase activities were measured using the luciferase assay system (Promega, Madison, WI, USA) as previously described [16]. Each experiment was performed in triplicate and repeated three times.

### Boyden chamber migration/invasion assay

Transwell assays were performed by using transwell plates (Corning). Cells were plated into the upper chamber with serum- and growth factor-free medium. The lower chamber was filled with serum-containing medium. After 24 h incubation at 37 °C, the cells on the upper chamber were removed. Cells that migrated to the lower side were fixed in 4% paraformaldehyde and stained with 0.1% crystal violet. The number of cells that migrated was counted at × 200 magnification in 10 different fields. The results were determined from three repeated experiments.

### Xenograft tumor metastasis model

For in vivo tumor metastasis experiment, a total of 1 × 10^6^ stable transfected cells suspended in 150 μl PBS were inoculated into the tail vein of nude mice, respectively. After 32 days housing, the lungs and livers of mice were dissected for histological examination (IHC and H&E staining). The images of the positive areas were taken. All experiments on mice were approved by the Institutional Animal Care and Use Committee of Third Military Medical University, China.

### WB analysis and immunohistochemistry (IHC)

WB was performed as previously described [17]. ACTIN was used as a loading control. The following primary antibodies were used: Tac2-N rabbit polyclonal antibody (1:500; Abcam), p65 rabbit polyclonal antibody (1:1000; Abcam), p-p65^S536^ rabbit polyclonal antibody (1:1000; Abcam), p50 rabbit polyclonal antibody (1:1000; Abcam), p-p50^S337^ rabbit polyclonal antibody (1:1000; Abcam), IκBα rabbit polyclonal antibody (1:1000; Abcam), p- IκBα^S32^ rabbit polyclonal antibody (1:1000; Abcam), p- IκBα^S36^ rabbit polyclonal antibody (1:1000; Abcam), beta-Tubulin rabbit polyclonal antibody (1:500, Bioss), Lamin A/C rabbit polyclonal antibody (1:500, Bioss) and ACTIN monoclonal antibody (1:2000; Sigma).

IHC staining was performed using a rabbit monoclonal antibody against Vimentin (1:500; Abcam) as described previously [12]. The vimentin staining was used to identify tumor cells in mice lungs and was also performed on paraffin-embedded tissues. The number and the size of metastatic foci were evaluated and quantified using vimentin staining.

### Gene set enrichment analysis (GSEA)

The datasets analyzed during the current study are available in the: https://xena.ucsc.edu/. To access the data please select “TCGA lung cancer” data set. In the second section “Select Your First Variable”, select “Genomic” for the data type and input the gene name “TC2N” then select Gene expression. The RNA-Seq data of 1020 NSCLC patients were retained and further analyzed.

GSEA is a computational method that determines whether an a priori defined set of genes shows statistically significant, concordant differences between two biologic states. TCGA data can be ordered in a ranked list, according to their differential expression between the phenotype. Tac2-N expression level was divided into low and high categories to annotate phenotype, and gene sets from the c5.mf.v6.2.symbols.gmt (curated) were used.

### Statistical analysis

Statistical analyses were performed with the SPSS 16.0 software (SPSS, Inc., Chicago, IL, USA). Each experiment was performed at least three times. The data were presented as the means ± SD. Student’s t-test or Mann-Whitney U test was used to compare means between groups. Cox regression models were used to analyze independent prognostic factors. Correlation analysis of gene expression was performed using Spearman’s rank correlation coefficient analysis. A two-sided *P*-value<0.05 was taken as statistically significant.

## Results

### The expression of Tac2-N is associated with tumor metastasis in lung cancer

**Fig. 1:**
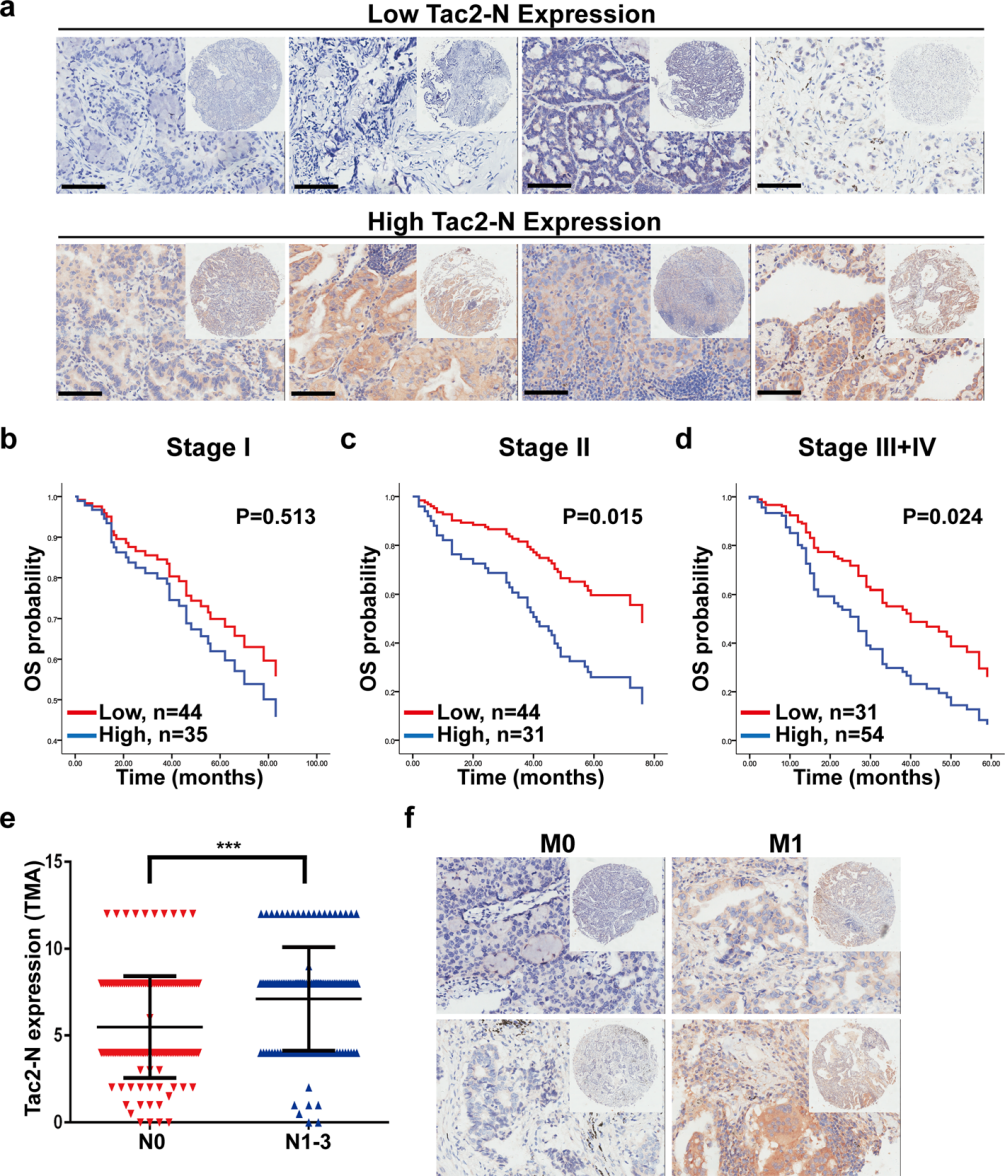
Tac2-N is associated with tumor metastasis of lung cancer patients. **a** The protein expression of Tac2-N was observed by IHC staining in lung cancer tissues. High Tac2-N expression group contains the ≥8 score patients. Low Tac2-N expression group contains the <8 score patients. Scale bars represent 50 μm. **b** Survival analysis of Tac2-N expression in I stage lung cancer patients by multivariate Cox regression analysis. **c** Survival analysis of Tac2-N expression in II stage lung cancer patients by multivariate Cox regression analysis. **d** Survival analysis of Tac2-N expression in III + IV stage lung cancer patients by multivariate Cox regression analysis. **e** The expression of Tac2-N was obviously higher in lung cancer patients with lymph node metastasis (N_1–3_) than that without lymph node metastasis (N_0_). ***P < 0.001. **f** The expression of Tac2-N was upregulated in lung cancer patients with metastasis than that without metastasis

In previous studies, we showed that Tac2-N expression correlates to clinical stages [15]. Therefore, we speculate its prognostic significance in different clinical stages are different. To evaluate the clinical implication of Tac2-N in different clinical stages, the tissue microarray contains 272 lung cancer lung cancer specimens were probed by IHC and then divided into two groups: low Tac2-N expression (scores< 8) and high Tac2-N (scores ≥8) (Fig. 1a). Subsequently, the associations of Tac2-N expression and the survival of patients with different clinical stages were analyzed by cox multivariate regression analysis. Results indicated that high Tac2-N expression was correlated with poor prognosis of II, III and IV stage patients, but not of I stage patients (Fig. 1b-d, Table 1). Tac2-N expression is associated with advanced stages instead of early stages, suggesting that Tac2-N may be correlated with malignant progression of lung cancer, such as tumor metastasis. Analysis of Tac2-N expression in patients with or without lymph nodal metastasis, we found that the expression of Tac2-N was obviously enhanced in the patients with lymph nodal metastasis (N_1–3_) compared to patients without lymph nodal metastasis (N_0_). (Fig. 1e, Table 2). Furthermore, Tac2-N was overexpressed in metastatic specimens compared with non-metastatic specimens (Fig. 1f).

**Table 1:** Multivariate analysis of different prognostic factors in different clinical stages

Variables	Clinical Stage					
	Stage I		Stage II		Stage III + IV	
	HR	P	HR	P	HR	P
TC2N expression	1.299	0.513	2.611	**0.015**	2.057	**0.024**
Age	2.400	0.059	2.380	0.050	0.870	0.602
Gender	1.090	0.835	1.250	0.600	1.348	0.352
Histological type	0.475	0.081	0.406	**0.045**	0.228	**< 0.001**
Histological grade	1.925	0.124	0.515	0.062	1.599	0.105
Tumor size	1.784	0.154	1.460	0.370	1.322	0.409

Bolded values indicate statistical significance, *P* < 0.05

Abbreviations: *HR* hazard ratio

**Table 2:** Association of TC2N expression with the status of tumor and lymph node

Variable	Category	Relative TC2N expression		*P*
		High (*n* = 131)	Low (*n* = 123)	
Depth of tumor invasion	T1-T2	98	95	0.651
	T3-T4	33	28	
Lymph node metastasis	N0	57	87	**< 0.001**
	N1–3	86	42	

Bolded values indicate statistical significance, *P* < 0.05

### Overexpression of Tac2-N promotes migration and invasion of lung cancer cells in vitro

**Fig. 2:**
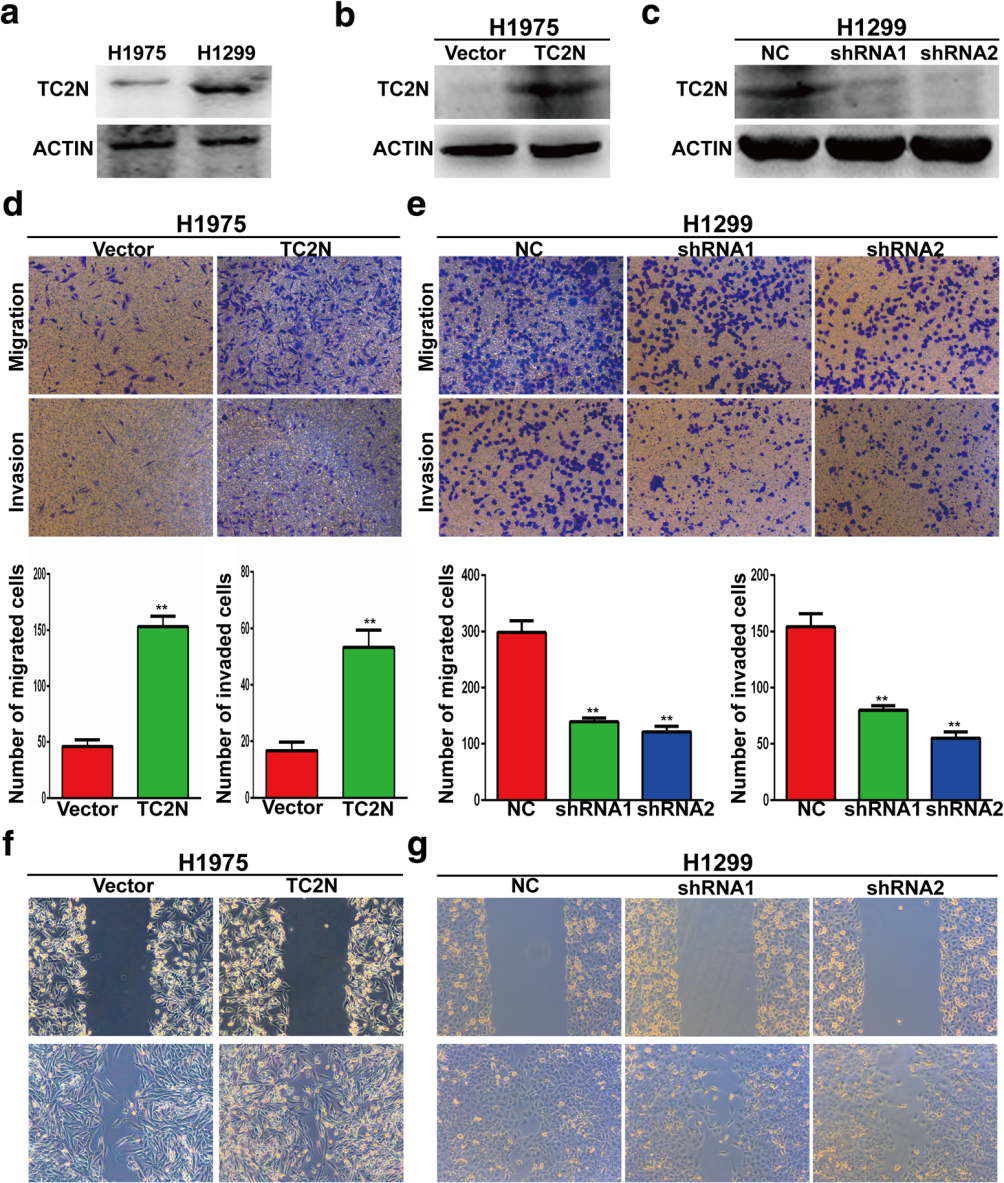
Tac2-N promotes migration and invasion of lung cancer cells in vitro. **a** The protein expression of Tac2-N was detected by WB. **b**, **c** Overexpression or knockdown of Tac2-N expression in two lung cancer cell lines were examined by WB. **d**, **e** Effects of Tac2-N enforced or silencing expression on migration and invasion of lung cancer cells detected by transwell assays. Mean ± S.D. (*n* = 3). **f**, **g** Representative images of wound healing in Tac2-N-overexpressed or -knockdown cells as compared with their controls. **P < 0.01

Given that Tac2-N overexpression was associated with advanced stages and lymph nodal metastasis, we continued to investigate whether Tac2-N could influence the migration and invasion of lung cancer cells. The Tac2-N was overexpressed in lung cancer H1975 cells (low expression of endogenous Tac2-N) or knocked-down in lung cancer H1299 cells (high expression of endogenous Tac2-N), and the expression of Tac2-N was verified by WB (Fig. 2a-c). Subsequently, transwell assay were performed to evaluate the migratory and invasive capacity of lung cancer cells after Tac2-N ectopic expression. Our data showed that Tac2-N overexpression promoted the migration and invasion of H1975 cells, whereas knockdown of Tac2-N inhibited the migration and invasion of H1299 cells (Fig. 2d and e). In addition, the wound-healing assay revealed the accelerated wound closure of Tac2-N-overexpressed lung cancer cells (Fig. 2f and g).

### Tac2-N overexpression enhances metastasis of lung cancer cells in vivo

**Fig. 3:**
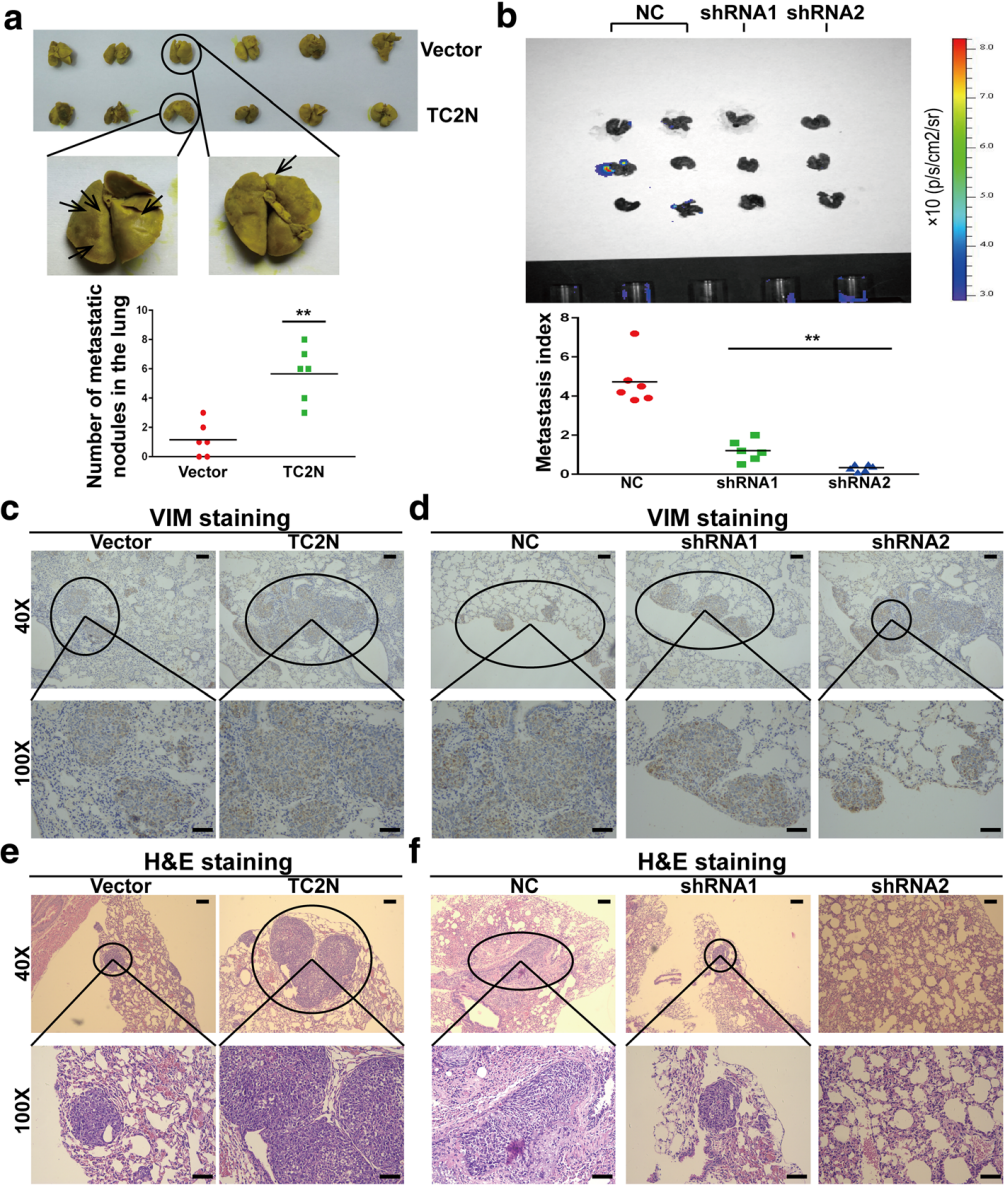
Tac2-N accelerates in vivo metastasis of lung cancer. **a** Lungs were removed from mice injected with H1975 cells stably expressing vector control or Tac2-N and fixed with Bouin’s solution. The photograph of lungs were taken. **b** Lungs were removed from mice injected with H1299 cells stably expressing negative control or Tac2-N shRNA. The fluorescent images of lung tissues from nude mice were photographed. **c**, **d** IHC evaluation of vimentin in lung necropsies and quantification of metastatic deposits. Scale bar represent 100 μm. **e**, **f** Lung invaded by tumor cells were further confirmed by H&E staining. Scale bar represent 100 μm

The above in vitro results encouraged us to evaluate the role of Tac2-N on tumor metastasis in vivo. H1975 cells with Tac2-N stable expression or H1299 cells with Tac2-N knockdown were injected into tail vein of nude mice, respectively. Noticeably, overexpression of Tac2-N resulted in a significant increase in the number and size of lung metastasis, while knockdown of Tac2-N reduced lung metastasis of H1299 cells (Fig. 3a and b). For a more direct assessment of metastatic potential, lung metastatic nodules of mice were further observed by IHC staining using human vimentin antibody and H&E staining. Consistently, the results revealed that cells with Tac2-N enforced expression produced more frequent and larger metastatic foci compared to control cells (Fig. 3c-f). Moreover, in order to further evaluate the function of Tac2-N in vivo, we examined the liver metastasis of nude mice using H&E staining and found that Tac2-N overexpression dramatically increased the number of metastatic foci in the liver of nude mice (Additional file 1: Figure S1a and S1b) and above results were further confirmed by detecting human-specific β2-MG (beta-2-microglobulin) levels to quantify metastatic human cancer cells (Additional file 1: Figure S1c and S1d). Collectively, these data together with the aforementioned results from in vitro assays suggested that Tac2-N is required to sustain the invasive and metastatic capacity of lung cancer cells.

### The activity of NF-κB signaling pathway is medicated by Tac2-N in lung cancer cells

**Fig. 4:**
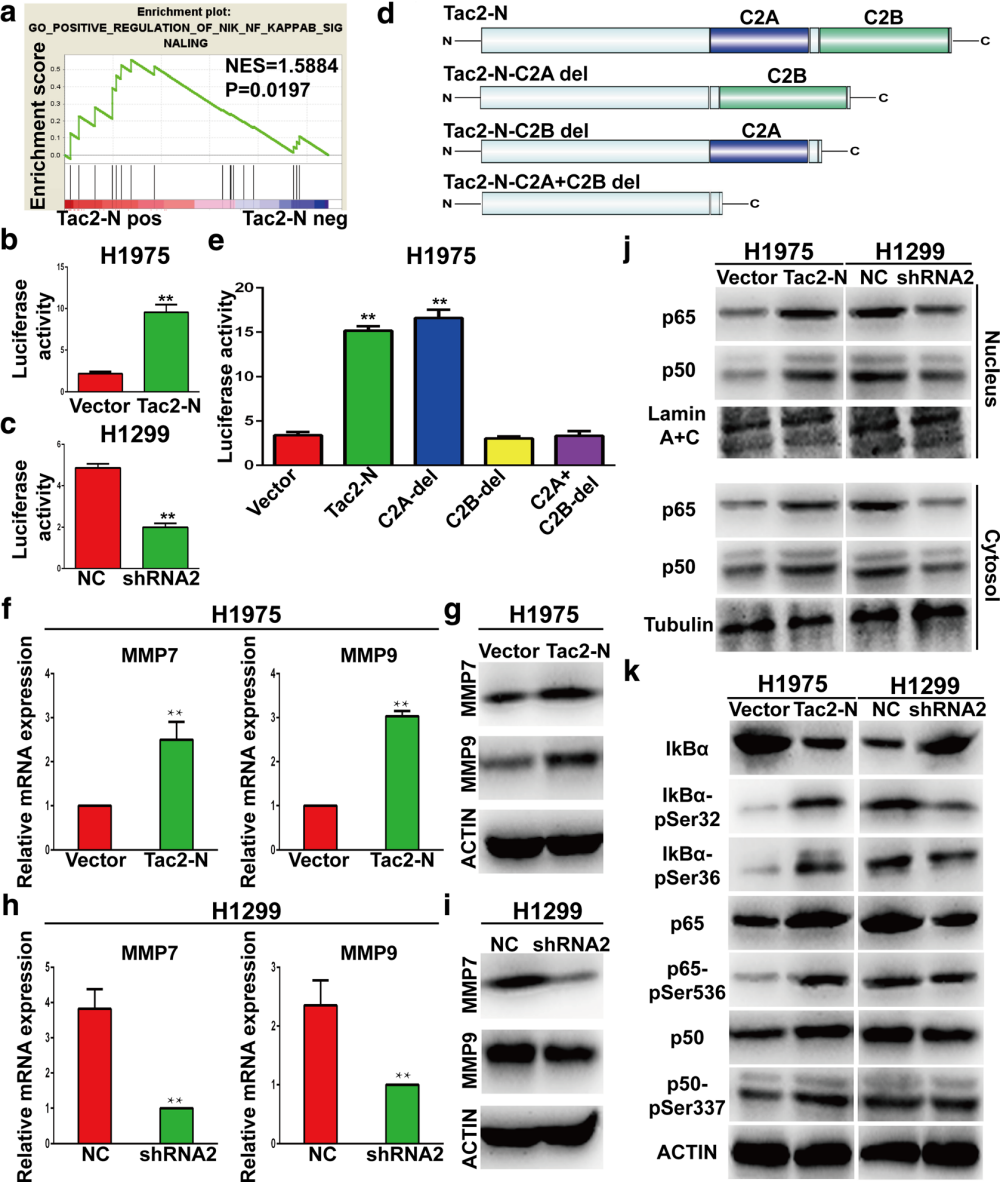
Tac2-N activates NF-κB signaling pathway in lung cancer cells. **a** GSEA showed that high Tac2-N expression was positively correlated with NF-κB signaling in lung cancer. **b** H1975 cells were co-transfected with vector control or Tac2-N expression vector and NF-kB reporter plasmid. The activity of NF-κB was measured at 24 h after the transfection. **c** H1299 cells were co-transfected with negative control or Tac2-N shRNA and NF-kB reporter plasmid. The activity of NF-κB was measured at 24 h after the transfection. **d** Schematic illustration of full-length and truncated Tac2-N constructs. **e** H1975 cells were co-transfected with vector control or Tac2-N expression vector or truncation constructs and NF-kB reporter plasmid. The activity of NF-κB was measured at 24 h after the transfection. **f** qRT-PCR analysis of MMP7 and MMP9 expression in H1975 cells transiently transfected with the vector control or Tac2-N. ACTIN was used as an internal control. **g** The protein expression of MMP7 and MMP9 were detected by WB in H1975 cells. **h** qRT-PCR analysis of MMP7 and MMP9 expression in H1299 cells transiently transfected with the negative control or Tac2-N shRNA. ACTIN was used as an internal control. **i** The protein expression of MMP7 and MMP9 were detected by WB in H1299 cells. **j** p65 and p50 protein levels in nucleus and cytoplasm were analyzed by WB. **k** WB analysis showed that Tac2-N could upregulation phosphorylation level of IκB with downregulation expression of IκB. ACTIN was used as an internal control

To uncover the downstream signaling pathway by which Tac2-N regulates metastasis phenotype in lung cancer, we performed Gene Set Enrichment Analysis (GSEA) using TCGA lung cancer dataset and found that NF-κB signaling pathway was enriched in this dataset (Fig. 4a). Accumulating evidence suggests that NF-κB signaling pathway contributes to metastasis of various types of cancer [18, 19]. To explore the possible involvement of Tac2-N in regulating NF-κB signaling pathway, we monitored NF-κB activation by measuring pNF-κB-luc-reporter vector expression in transiently transfected H1975 cells or H1299 cells. Results showed that there was a significant increase in NF-κB activity after Tac2-N overexpression, while Tac2-N silencing remarkably suppressed the activity of NF-κB (Fig. 4b and c). Further, we investigated which domain of Tac2-N is involved in regulation of NF-κB signaling pathway. H1975 cells were transfected with wild-type (full-length) Tac2-N or one of a panel of deletion, or truncation constructs, including Tac2-N-del-1 (lack C2A domain of Tac2-N), Tac2-N-del-2 (lack C2B domain of Tac2-N) and Tac2-N-del-3 (lack C2A and C2B domain of Tac2-N) in expression vectors (Fig. 4d). Luciferase reporter experiments revealed that full-length Tac2-N and deletion 1 Tac2-N but not deletion 2 Tac2-N and deletion 3 Tac2-N activated NF-κB (Fig. 4e). This result demonstrates that the C2B domain of Tac2-N is necessary for regulation of NF-κB. Then qRT-PCR and WB analysis were performed to measure the expression changes of NF-κB signaling pathway target genes in H1975 and H1299 cells. Consistent with the results of luciferase reporter assay, the expression of targets were upregulated in H1975 cells with Tac2-N transfection (Fig. 4f and g), and were downregulated in H1299 cells with Tac2-N knockdown (Fig. 4 h and i).

### Tac2-N increases the translocation of NF-κB to the nucleus of lung cancer cells through promoting phosphorylation of IκB

The level of nuclear NF-κB, which is an indicator of active NF-κB signaling pathway contributes to activate the transcription of target gene [20]. Thus, we detected the expression and distribution of NF-κB between nucleus and cytoplasm. The WB results showed that the expression of NF-κB in both nucleus and cytoplasm were significantly elevated in H1975 cells with Tac2-N overexpression, whereas it was dramatically decreased in H1299 cells with Tac2-N knockdown, compared with their negative control cells (Fig. 4j). Next, we focused on elucidating the molecular mechanism of NF-κB transportation to the nucleus that regulated by Tac2-N. Notably, the IκB acts as a gatekeepers, limit NF-κB migration into the nucleus and mask its DNA-binding and nuclear localization domains [5]. Therefore, WB assays were used to examine whether Tac2-N regulates the phosphorylation of IκB. Indeed, the overexpression of Tac2-N upregulated the phosphorylation level of IκB in H1975, whereas knockdown of Tac2-N significantly reduced IκB phosphorylation in H1299 cells (Fig. 4k).

### The oncogenic function of Tac2-N is dependent on NF-κB signaling pathway

**Fig. 5:**
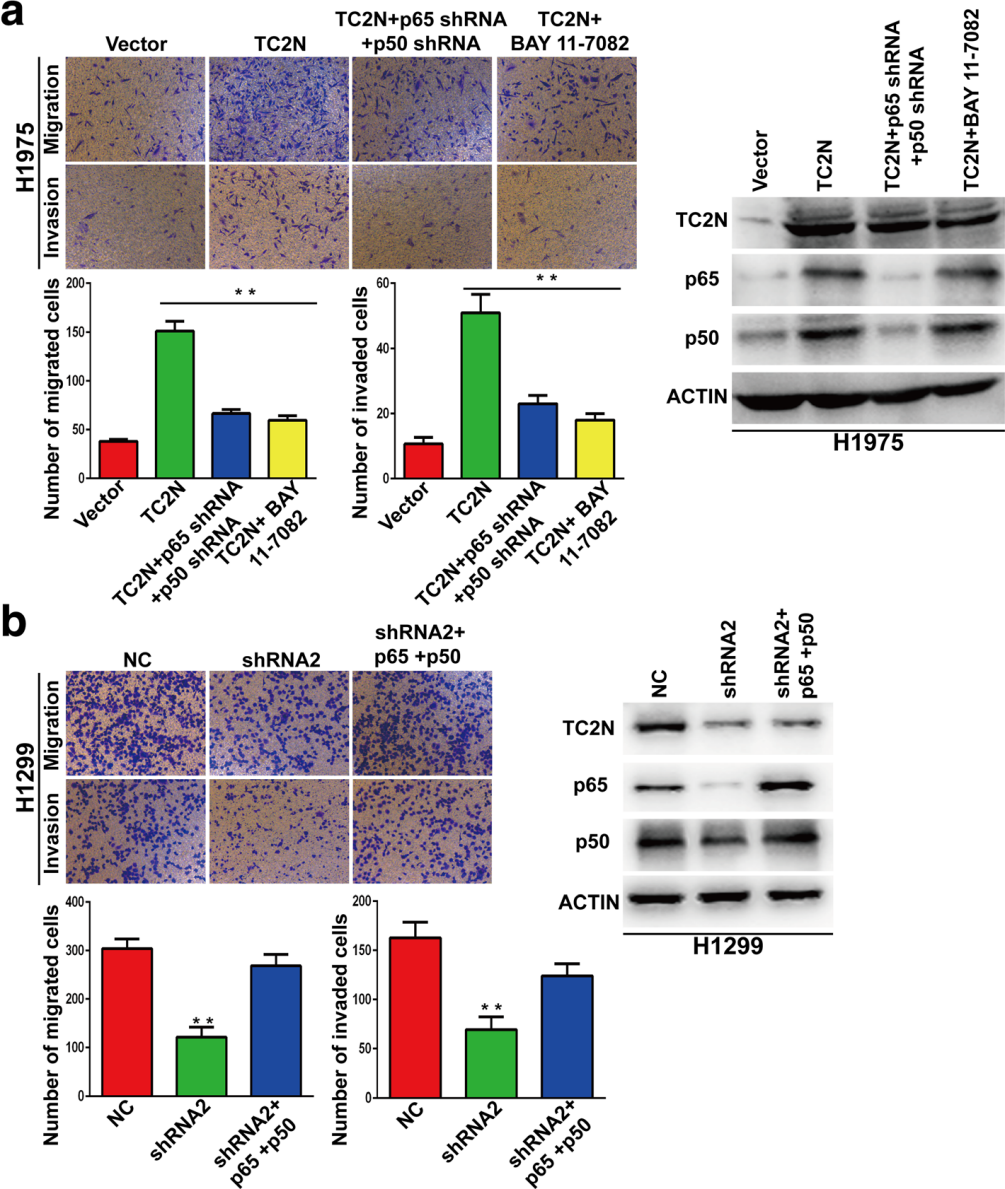
NF-κB signaling pathway is indispensable for Tac2-N-medicated lung cancer metastasis. **a** Transwell assays were used to examine the effect of NF-κB ablation on cell metastasis of H1975-Tac2-N cells. **b** Transwell assays were used to examine the effect of overexpression of p65 and p50 on cell metastasis of Tac2-N-silencing H1299 cells

Encouraged by the above results, we next explore the metastasis-promoted effect of Tac2-N is due to its ability to act as an activator of NF-κB signaling pathway. The activity of NF-κB was blocked by using siRNA or inhibitor BAY 11–7082 when overexpression of Tac2-N. We found that Blockade of NF-κB did not markedly inhibit the migration and invasion of Tac2-N-overexpressing cells, while it significantly inhibited the migration and invasion of cells transfected with vector control (Fig. 5a and b). Taken together, these data suggested that Tac2-N promoted metastasis of lung cells in a NF-κB signaling pathway dependent manner.

## Discussion

Lung cancer patients with poor outcome greatly depend on tumor metastasis. Thus, human desperately need to elucidate the molecular mechanisms of tumor metastasis. Tac2-N has been recently identified as a novel oncogene by promoting tumor growth of lung cancer. In this study, to further explore the precise role of Tac2-N in lung cancer, we identified for the first time the association between Tac2-N expression and different clinical stages in lung cancer. The results showed that the expression of Tac2-N was associated with poor outcome of advanced stage patients, but not of early stage patients, indicating that there may be a relation between Tac2-N expression and tumor progression. Indeed, further studies testified that Tac2-N expression is linked to metastatic phenotype of lung cancer and upregulation of Tac2-N promotes migration and invasion of lung cancer cells in vitro, and tumor metastasis in vivo. This means that Tac2-N serves as a typical metastatic promoter in lung cancer.

The NF-κB transcription factor family has been considered the central mediator of the inflammatory process and a key participant in tumor progression [21]. There is a close relationship between excessive expression and activate of NF-κB and the occurrence, development and the malignant biological behavior of the cancer [22]. Furthermore, NF-κB has been reported as critical for regulation of tumor cell metastasis and epithelial mesenchymal transition (EMT) [6, 23]. EMT has been thought to constitute the core of embryogenesis for several decades, has recently been shown to have a critical role in controlling of tumor progression, such as invasion and metastasis [23–25]. After undergoing EMT program, epithelial tumor cells could acquire enhanced invasive and metastatic traits, which was associated with high-grade malignancy [26, 27]. NF-κB family has been identified as a central mediator of EMT. Multiple lines of evidence indicate that EMT-related transcription factors are regulated directly or indirectly by NF-κB [23]. In our study, we demonstrated that Tac2-N enhanced the metastasis of lung cancer through activating the NF-κB signaling pathway. Does that mean Tac2-N could also regulate EMT in lung cancer? This hypothesis should be empirically tested, and further studies are required to address this aim. Further study showed that C2B domain not C2A domain of Tac2-N is essential to regulate NF-κB signaling pathway in lung cancer cells. Due to the fact that the C2B domain of Tac2-N is required for transporting it into the nucleus [14], we suspect the transportation of Tac2-N to the nucleus contributes to Tac2-N performs its functions.

Moreover, Tac2-N-induced activation of NF-κB signaling pathway in lung cancer cells was found to be carried out by inducing IκB phosphorylation with leading to increase nuclear translocation of the NF-κB. It needs to be pointed out, the upregulation of NF-κB upon Tac2-N overexpression occurred on a posttranscriptional level, because NF-κB protein levels but not mRNA levels were increased (Fig. 4k, Additional file 1: Figure S2) following overexpression of Tac2-N. Further studies are required to determine this molecular mechanism.

## Conclusion

In conclusion, we have identified Tac2-N as a novel therapeutic target and novel biomarker, exerted a pivotal role in promoting metastasis via NF-κB signaling pathway in lung cancer. Moreover, our data predicted the application of NF-κB inhibitors for precision therapy of lung cancer with elevated Tac2-N expression.

## Additional file

Additional file 1: **Figure S1.** TC2N promotes lung and liver metastasis of lung cancer cells in nude mice. **a**, **b** Liver metastases were observed by H&E staining. **c, d** Lung and liver metastasis were further quantified using RT-qPCR. Human-specific β2-MG levels were used to quantify metastatic human cancer cells with the mouse-specific β2-MG level as an internal control. Error bars indicate s.d. (*n* = 4).*P < 0.05. **Figure S2.** Ectopic expression of TC2N does not affected the NF-κB mRNA expression. **a** The mRNA expression of p65 and p50 were examined by qRT–PCR after reexpression of TC2N in H1975 cells. **b** The mRNA expression of p65 and p50 were examined by qRT–PCR after knockdown of TC2N in H1299 cells. ACTIN serves as an internal control. **Table S1.** The primer applied in the study. (ZIP 1723 kb)

## Data Availability

The datasets analyzed during the current study are available in the: https://xena.ucsc.edu/.

## Abbreviations

IHC: Immunohistochemistry
TMA: Tissue microarray
WB: Western Blot
β2-MG: beta-2-microglobulin

## References

[1] Torre LA, Bray F, Siegel RL, Ferlay J, Lortettieulent J, Jemal A (2015). Global cancer statistics, 2012. CA Cancer J Clin.

[2] Molina JR, Yang P, Cassivi SD, Schild SE, Adjei AA (2008). Non–small cell lung Cancer: epidemiology, risk factors, treatment, and survivorship. Mayo Clin Proc.

[3] Roy SS, Gonugunta VK, Bandyopadhyay A, Rao MK, Goodall GJ, Sun LZ (2014). Significance of PELP1/HDAC2/miR-200 regulatory network in EMT and metastasis of breast cancer. Oncogene..

[4] Colomer C, Marruecos L, Vert A, Bigas A, Espinosa L (2017). NF-Î^o^B members left home: NF-Î^o^B-independent roles in Cancer. Biomedicines..

[5] Didonato JA, Mercurio F, Karin M (2012). NF-kB and the link between inflammation and cancer. Immunol Rev.

[6] Rinkenbaugh Amanda, Baldwin Albert (2016). The NF-κB Pathway and Cancer Stem Cells. Cells.

[7] Cho W, Stahelin RV (1761). Membrane binding and subcellular targeting of C2 domains. Biochim Biophys Acta Mol Cell Biol Lipids.

[8] Corbalangarcia S, Gómezfernández JC (2014). Signaling through C2 domains: more than one lipid target. Biochim Biophys Acta.

[9] Duncan RR (2000). Double C2 protein. A review Biochimie.

[10] Farah CA, Sossin WS (2012). The role of C2 domains in PKC signaling. Adv Exp Med Biol.

[11] Li H, Xiao N, Wang Y, Wang R, Chen Y, Pan W, Liu D, Li S, Sun J, Zhang K, Sun Y, Ge X (2017). Smurf1 regulates lung cancer cell growth and migration through interaction with and ubiquitination of PIPKIγ. Oncogene.

[12] Blomme A, Costanza B, De TP, Thiry M, Van SG, Boutry S (2017). Myoferlin regulates cellular lipid metabolism and promotes metastases in triple-negative breast cancer. Oncogene..

[13] Tanksley JP, Chen X, Coffey RJ (2013). NEDD4L is downregulated in colorectal Cancer and inhibits canonical WNT signaling. PLoS One.

[14] Fukuda M, Mikoshiba K (2001). Tac2-N, an atypical C-type tandem C2 protein localized in the nucleus. FEBS Lett.

[15] Hao XL, Han F, Zhang N, Chen HQ, Jiang X, Yin L, Liu WB, Wang DD, Chen JP, Cui ZH, Ao L, Cao J, Liu JY (2018). TC2N, a novel oncogene, accelerates tumor progression by suppressing p53 signaling pathway in lung cancer.

[16] Han Fei, Liu Wen-bin, Shi Xiao-yan, Yang Jun-tang, Zhang Xi, Li Zhi-ming, Jiang Xiao, Yin Li, Li Jian-jun, Huang Chuan-shu, Cao Jia, Liu Jin-yi (2018). SOX30 Inhibits Tumor Metastasis through Attenuating Wnt-Signaling via Transcriptional and Posttranslational Regulation of β-Catenin in Lung Cancer. EBioMedicine.

[17] Zhao F, Wang M, Li S, Bai X, Bi H, Liu Y (2015). DACH1 inhibits SNAI1-mediated epithelial–mesenchymal transition and represses breast carcinoma metastasis. Oncogenesis..

[18] Lee CH, Jeon YT, Kim SH, Song YS (2007). NF-kappaB as a potential molecular target for cancer therapy. Biofactors..

[19] Aggarwal BB (2004). Nuclear factor-kappaB: the enemy within. Cancer Cell.

[20] Chaturvedi M M, Sung B, Yadav V R, Kannappan R, Aggarwal B B (2010). NF-κB addiction and its role in cancer: ‘one size does not fit all’. Oncogene.

[21] Xia Y, Shen S, Verma IM (2014). NF-κB, an active player in human cancers. Cancer Immunol Res.

[22] Ml DCB, Da CR, Fraga AG, Camarinha BD, Gc DCS, Lima AG, et al. NF-κB signaling pathway inhibitors as anticancer drug candidates. Anti-Cancer Agents Med Chem Former Curr Med Chem - Anti-Cancer Agents. 2016;17. 10.2174/1871520616666160729112854 27481554

[23] Min C, Eddy SF, Sherr DH, Sonenshein GE (2008). NF-kappaB and epithelial to mesenchymal transition of cancer. J Cell Biochem.

[24] Singh Mohini, Yelle Nicolas, Venugopal Chitra, Singh Sheila K. (2018). EMT: Mechanisms and therapeutic implications. Pharmacology & Therapeutics.

[25] Jiang ZS, Sun YZ, Wang SM, Ruan JS (2017). Epithelial-mesenchymal transition: potential regulator of ABC transporters in tumor progression. J Cancer.

[26] Thiery JP, Sleeman JP (2006). Complex networks orchestrate epithelial-mesenchymal transitions. Nat Rev Mol Cell Biol.

[27] Yang J, Weinberg RA (2008). Epithelial-mesenchymal transition: at the crossroads of development and tumor metastasis. Dev Cell.

