# Supplementary figures and images for: Tac2-N acts as a novel oncogene and promotes tumor metastasis via activation of NF-κB signaling in lung cancer
Source: J Exp Clin Cancer Res. 2019 Aug 30;38:319. doi: 10.1186/s13046-019-1316-7 (PMC6716936; doi:10.1186/s13046-019-1316-7)

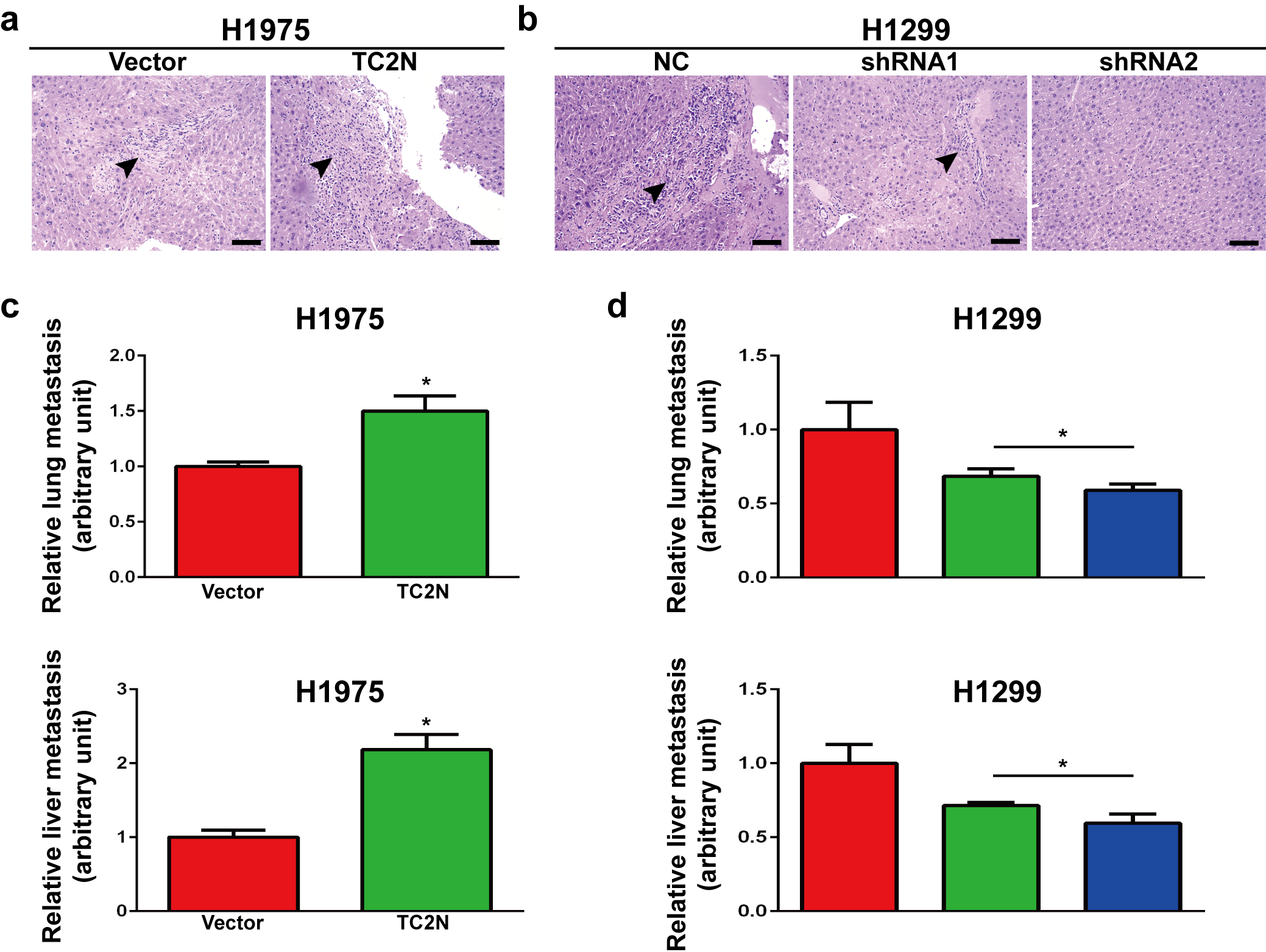

Supplement: Supplementary file 1 — Figure S1. TC2N promotes lung and liver metastasis of lung cancer cells in nude mice. a, b Liver metastases were observed by H&E staining. c, d Lung and liver metastasis were further quantified using RT-qPCR. Human-specific β2-MG levels were used to quantify metastatic human cancer cells with the mouse-specific β2-MG level as an internal control. Error bars indicate s.d. (n = 4).*P < 0.05. Figure S2. Ectopic expression of TC2N does not affected the NF-κB mRNA expression. a The mRNA expression of p65 and p50 were examined by qRT–PCR after reexpression of TC2N in H1975 cells. b The mRNA expression of p65 and p50 were examined by qRT–PCR after knockdown of TC2N in H1299 cells. ACTIN serves as an internal control. Table S1. The primer applied in the study. (ZIP 1723 kb) [file 13046_2019_1316_MOESM1_ESM.zip › S1.tif]

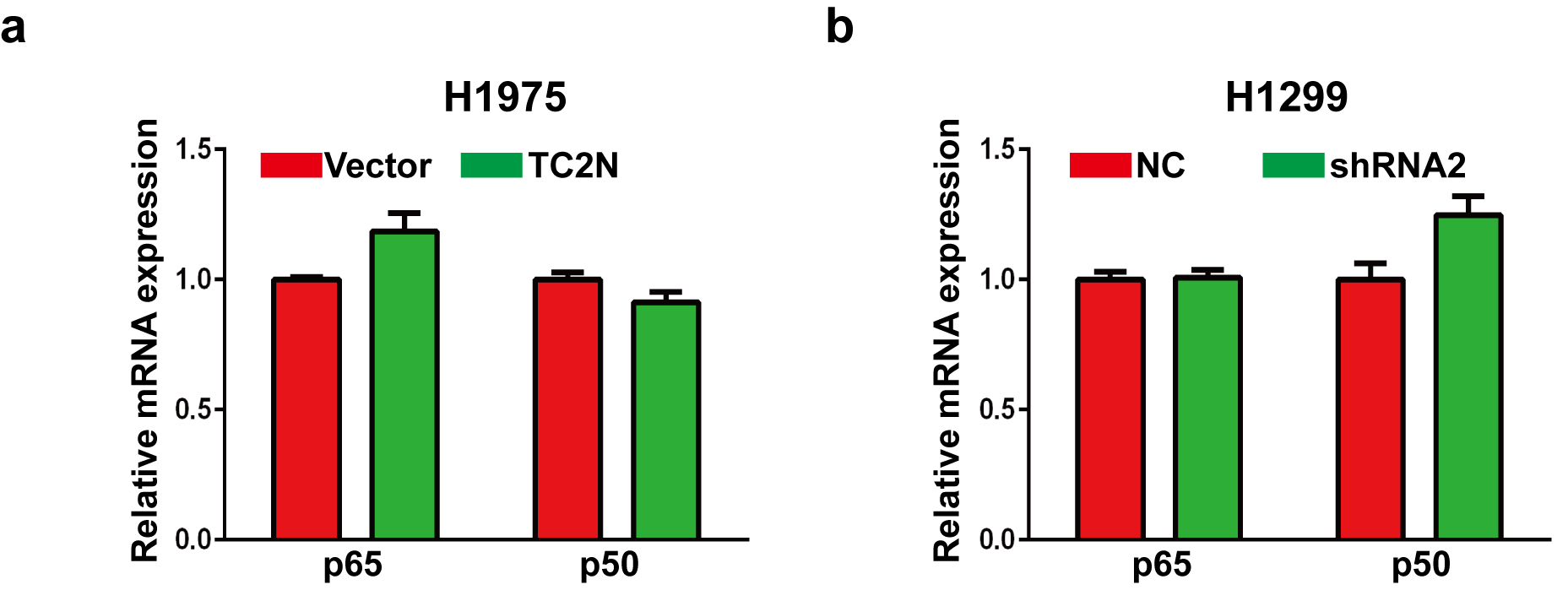

Supplement: Supplementary file 1 — Figure S1. TC2N promotes lung and liver metastasis of lung cancer cells in nude mice. a, b Liver metastases were observed by H&E staining. c, d Lung and liver metastasis were further quantified using RT-qPCR. Human-specific β2-MG levels were used to quantify metastatic human cancer cells with the mouse-specific β2-MG level as an internal control. Error bars indicate s.d. (n = 4).*P < 0.05. Figure S2. Ectopic expression of TC2N does not affected the NF-κB mRNA expression. a The mRNA expression of p65 and p50 were examined by qRT–PCR after reexpression of TC2N in H1975 cells. b The mRNA expression of p65 and p50 were examined by qRT–PCR after knockdown of TC2N in H1299 cells. ACTIN serves as an internal control. Table S1. The primer applied in the study. (ZIP 1723 kb) [file 13046_2019_1316_MOESM1_ESM.zip › S2.tif]
